# Directivity and Excitability of Ultrasonic Shear Waves Using Piezoceramic Transducers—Numerical Modeling and Experimental Investigations

**DOI:** 10.3390/s24113462

**Published:** 2024-05-27

**Authors:** Emil Aleksiewicz-Drab, Aleksandra Ziaja-Sujdak, Rafał Radecki, Wiesław J. Staszewski

**Affiliations:** 1 Wave Propagation and Signal Processing (WPSP), Department of Physics, KU Leuven—Campus Kulak, 8500 Kortrijk, Belgium; emil.aleksiewicz-drab@kuleuven.be; 2 AGH University of Krakow, Faculty of Mechanical Engineering and Robotics, Department of Robotics and Mechatronics, Al. Mickiewicza 30, 30-059 Kraków, Poland; aleksandra.ziaja@agh.edu.pl (A.Z.-S.); rafal.radecki@agh.edu.pl (R.R.)

**Keywords:** shear horizontal waves, piezoceramic-based excitation, directivity, excitability, multi-sensor configuration, numerical simulations, experimental tests

## Abstract

In this paper, piezoceramic-based excitation of shear horizontal waves is investigated. A thickness-shear d_15_ piezoceramic transducer is modeled using the finite-element method. The major focus is on the directivity and excitability of the shear horizontal fundamental mode with respect to the maximization of excited shear and minimization of Lamb wave modes. The results show that the geometry of the transducer has more effect on the directivity than on the excitability of the analyzed actuator. Numerically simulated results are validated experimentally. The experimental results show that transducer bonding significantly affects the directivity and amplitude of the excited modes. In conclusion, when the selected actuator is used for shear excitation, the best solution is to tailor the transducer in such a way that at the resonant frequency the desired directivity is achieved.

## 1. Introduction

Smart structures with bonded/embedded sensors are attractive for Structural Health Monitoring (SHM) applications, particularly when guided ultrasonic waves are used for this task. Various approaches based on guided ultrasonic wave have been developed for damage detection [1,2,3]. This includes methods based on Lamb waves that have been investigated for the last few decades. Recent years have brought research interest in shear horizontal (SH) ultrasonic waves. In contrast to Lamb waves, the fundamental SH wave—known as SH_0_—is non-dispersive. Previous research studies also show that it is sensitive to small structural defects, particularly when the nonlinear wavefield is used [4,5,6,7,8,9]. Despite significant progress in this field, SH wave excitation still remains a challenge.

SH waves can be excited using Lorentz force-based, magneto-strictive and piezoelectric effects. Various types of single, array, omnidirectional transducer technologies have been developed for shear wave excitation and SHM applications [10]. Electro-Magnetic Acoustic Transducers (EMAT) utilize either the Lorentz force-based or magneto-strictive effects [11]. These transducers can excite and receive SH waves, but are non-contact or require coupling. In addition, these transducers are quite bulky and their efficiency for excitation/sensing is relatively low, leading to poor signal-to-noise ratios [10]. Magneto-Strictive Transducers (MSTs) patches utilize the magneto-strictive effect for excitation [12]. These transducers are thin and can be surface-bonded to monitored structures but their excitation area is relatively large. The piezoelectric effect is utilized for the excitation of SH waves in piezoelectric probes [13,14] and piezoelectric low-profile transducers such as PWASs (piezoelectric wafer active sensors) and PFPs (piezoelectric fiber patches) transducers [15,16]. The piezoelectric probe transducers are often used for material defect detection/monitoring in nondestructive testing (NDT) applications. The piezoelectric low-profile transducers are quite flexible and smaller than MST patches in terms of bonding. Therefore, these transducers are quite attractive for SH wave actuation and sensing in damage-detection SHM applications that require the permanent bonding of actuators/sensors on monitored structures.

PZT (lead–zirconate–titanate) ceramics are the most widely used piezoelectric materials for ultrasonic wave generation and sensing. Sensors based on PZT ceramics have been used for SHM applications for decades. More recently, a number of plate and stack actuators have been developed for SH wave generation. The latter are nowadays available commercially in various shapes, poling and electrode configurations. PZT ceramic shear wave actuators can operate in face-mode [17] and thickness-mode [18]. The face-shear transducers use the d_36_ and d_24_ coefficients. The generation action of the d_24_ mode transducer is very similar to the Wiedemann effect in magneto-strictive materials. Transducers operating in d_36_ mode also generate extensional d_31_ modes. The thickness-shear transducers use the d_15_ coefficient for shear wave generation. These transducers can be poled in the in-plane or through-thickness configurations. Thickness-shear mode transducers also generate unwanted Lamb waves that will make the signal-to-noise ratios of response relatively low. The work in [19,20] indicates that the amplitude of unwanted Lamb waves can be minimized by optimizing the geometry. Increasing the length of the d_15_ wafer will enhance the excited shear wave and suppress the unwanted Lamb waves. However, then PZT wafers become difficult for poling and unpractically large for surface-bonding. Face-shear transducers usually transfer energy into hosting structures more effectively than thickness-shear transducers. In addition, face-shear transducers are better for pure shear-mode excitation and offer better generation/reception properties. Thickness-shear transducers are more suitable for phase array systems based on omnidirectional shear waves. A good comparison of three different types of transducers—that favors the face-shear d_24_ transducer over thickness-shear d_15_ transducers—is given in [21].

The paper revisits a d_15_ thickness-shear mode PZT ceramic transducer that has been tested in previous research studies and judged unfavorably. This time, a *STEMiNC*^®^ shear-mode SM411 PZT ceramic transducer (STEINER & MARTINS, INC. 39873 US Highway 27, Suite 225 Davenport, FL 33837-7802–USA) is put into tests that involve modeling and experimental work related to directivity and excitability. There are two nickel electrodes placed on each side (S configuration) of this relatively small 6 × 6 × 2.5 mm transducer. As a consequence of this unusual configuration, one connection to the transducer needs to be made on its bottom surface that is bonded to a host structure.

In contrast to previous work in this field, shear wave generation is the major focus. Reception is performed using non-contact laser vibrometry. Multi-sensor configuration is also investigated, being an additional novelty. Three important questions are as follows: (1) Is this small, off-the-shelf, relatively cheap, unusually wired transducer still capable to generate shear horizontal? (2) Is the amplitude of the generated shear horizontal wave significantly higher than the amplitude of the additionally generated and unwanted Lamb waves? (3) What is the best excitation frequency for SHM application?

Research work is presented in this paper using five different sections. For the sake of completeness, Section 2 gives the theoretical background-related SH wae propagation. The physical and mathematical model of the investigated transducer is described in Section 3. Numerical simulation results are presented in Section 4. Experimental tests are described in Section 5. Finally, conclusions are provided in Section 6.

## 2. Shear Horizontal Waves

Particle wave motion can be described using the Navier’s displacement equations [22,23]

(1) $$\mu \nabla^{2}u\left(x,t\right)+\left(\lambda +\mu \right)\nabla \nabla u\left(x,t\right)=\rho \frac{\partial^{2}u(x,t)}{\partial t^{2}},$$

where the vector $u=\left[u_{1},u_{2},u_{3}\right]$ describes the displacement field vector, ρ is the density and λ, μ are the Lamé constants. The particle motion of shear waves is perpendicular to the wave propagation direction. These waves can be polarized horizontally and vertically. Therefore, the former are known as SH waves, whereas the latter as SV waves. The fundamental mode of SH—i.e., the SH_0_ wave mode—is particularly attractive for structural damage detection applications due to its non-dispersive nature and good sensitivity to micro-structural damage. For the SH_0_ particle motion description, Equation (1) can be simplified as [22,23]

(2) $$\frac{\partial^{2}u_{3}}{\partial x_{1}^{2}\;}+\frac{\partial^{2}u_{3}}{\partial x_{2}^{2}\;}=\frac{1}{c_{T}^{2}}\frac{\partial^{2}u_{3}}{\partial t^{2}\;},$$

where $u_{3}(x_{1},x_{2},t)$ is the only non-zero displacement component and $c_{T}$ is the bulk shear wave speed that can be given as $c_{T}^{2}=\mu /\rho .$ The wave propagates along the $x_{1}$ axis. The $x_{2}$ axis is vertical, while the $x_{1}$ and $x_{3}$ axes define the horizontal plane. The non-zero displacement component $u_{3}$ can be assumed as a harmonic function, i.e.,

(3) $$u_{3}\left(x_{1},x_{2},t\right)=f\left(x_{2}\right)e^{i\left(kx_{1}-\omega t\right)},$$

where ω is the circular frequency and k=2π/λ is the wavenumber. Substituting Equation (3) to Equation (2) yields

(4) $$\frac{\partial^{2}f\left(x_{2}\right)}{\partial x_{2}^{2}}+\left(\frac{\omega^{2}}{c_{T}^{2}}-k^{2}\right)f\left(x_{2}\right)=0.$$

The general solution of this equation can be given as

(5) $$f\left(x_{2}\right)=Asin\left(qx_{2}\right)+Bcos\left(qx_{2}\right),$$

where

(6) $$q=\sqrt{\frac{\omega^{2}}{c_{T}^{2}}-k^{2}},$$

and *A*, *B* are arbitrary amplitudes. For the plate of d=2h thickness, the upper and lower surface ($x_{2}=\pm h$) can be assumed as traction free. Then, the relevant traction force given as

(7) $$\tau_{23}\left(x_{1},\;x_{2},t\right){|}_{x_{2}=\pm h}=0,$$

is the only nontrivial boundary condition. By imposing this boundary condition on the components given in Equation (3), the characteristic equations can be obtained as

(8) $$\sin\left(qh\right)=0\;\mathrm{and}\;\cos\left(qh\right)=0,$$

for the symmetric and antisymmetric components, respectively. Since $\sin\left(x\right)=0$ when x=nπ(n∈{0,1,2,…}) and $\cos\left(x\right)=0$, when $x=n\pi /2(n\in \left\{1,3,5,\ldots \right\})$, the solutions to Equation (8) can be written as

(9) qh=nπ/2,

where $n\in \left\{0,2,4,\ldots \right\}$ for symmetric and $n\in \left\{1,3,5,\ldots \right\}$ for antisymmetric modes. As a result, the displacement given by Equation (3) can be separated into symmetric and antisymmetric components—with respect to $x_{2}$—as

(10) $$u_{3}^{s}\left(x_{1},x_{2},t\right)=B\cos\left(qx_{2}\right)e^{i\left(kx_{1}-\omega t\right)}\;\mathrm{and}\;u_{3}^{a}\left(x_{1},x_{2},t\right)=A\sin\left(qx_{2}\right)e^{i\left(kx_{1}-\omega t\right)},$$

respectively. When Equation (6) is considered, Equation (9) can be re-written as

(11) $$\frac{\omega^{2}}{c_{T}^{2}}-\frac{\omega^{2}}{c_{P}^{2}}={\left(\frac{n\pi }{2h}\right)}^{2}.$$

For ω=2πf, the above equation can be solved for the phase velocity $c_{P}$—with respect to the frequency-thickness (fd) product—to give

(12) $$c_{P}\left(fd\right)=\pm 2c_{T}\left\{\frac{fd}{\sqrt{4{\left(fd\right)}^{2}-n^{2}c_{T}^{2}}}\right\}.$$

The solution for *n =* 0 corresponds to the SH_0_ mode. One can note that in this case $c_{P}=c_{T}$ and the dispersionless wave propagates with the bulk shear wave speed $c_{T}$. By analogy, the relevant group velocity can be obtained as

(13) $$c_{g}\left(fd\right)=c_{T}\sqrt{1-\frac{{\left(n/2\right)}^{2}}{{\left(fd/c_{T}\right)}^{2}}}\;\;\;\;\;\;\;\;\;\;\;\;\;\;\left(fd\geq {\left(fd\right)}_{n}\right).$$

This clearly shows that at the cut-off frequencies—$f_{co}=\left(c_{T}q\right)/\left(2\pi \right)$—the group velocity of any mode is zero, and as fd  approaches infinity for any given fixed n, the group velocity of any SH mode approaches the velocity of bulk shear waves $c_{T}$. Equations (12) and (13) also show that the SH_0_ mode of interest in this paper is non-dispersive.

## 3. Piezoceramic Shear Wave Actuator and Wave Propagation Modeling

This section describes the model of the piezoceramic equation used for SH_0_ wave excitation. For the sake of completeness, the theoretical background related to piezoelectricity is provided. The piezoelectric equations are introduced and given for the transducer type used. Then, the finite-element model is described.

### 3.1. Piezoelectric Equations—Theoretical Background

**Figure 1:**
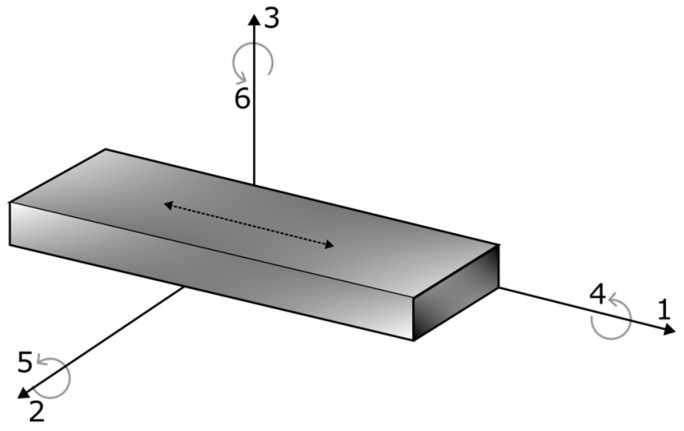
Tensor direction definitions used in the constitutive electro-mechanical equations.

It is well known that the piezoelectric effect relates to the cross-coupling of the mechanical field and electrical field. The electro-mechanical equations associated with this effect can be given as below [24].

(14) $$\left\{S\right\}=\left[s^{E}\right]\left\{T\right\}+\left[d^{T}\right]\{E\}$$

(15) $$\left\{D\right\}=\left[d\right]\left\{T\right\}+\left[\varepsilon^{T}\right]\{E\}$$

where *S* is the strain tensor, *T* is stress tensor, *E* is the electric field strength tensor, *D* is the electric flux density tensor, s is the compliance under short-circuit condition, ε is the permittivity and d and $d^{T}$ are the matrices for the direct and converse piezoelectric effect, respectively. The superscripts *T* and *E* in Equations (14) and (15) indicate the constant (or zero) stress and electric field, respectively. The tensor directions are defined in Figure 1. One should note that “1”, “2” and “3” describes the longitudinal, transverse and thickness directions, respectively. The shear planes are denoted by the “4”, “5” and “6” subscripts.

Following the definitions provided by Figure 1, Equations (14) and (15) can be re-written and expanded as

(16) $$\left[\begin{array}{c} S_{1}\\ S_{2}\\ S_{3}\\ S_{4}\\ S_{5}\\ S_{6} \end{array}\right]=\left[\begin{array}{cccccc} S_{11}^{E} & S_{12}^{E} & S_{13}^{E} & S_{14}^{E} & S_{15}^{E} & S_{16}^{E}\\ S_{21}^{E} & S_{22}^{E} & S_{23}^{E} & S_{24}^{E} & S_{25}^{E} & S_{26}^{E}\\ S_{31}^{E} & S_{32}^{E} & S_{33}^{E} & S_{34}^{E} & S_{35}^{E} & S_{36}^{E}\\ S_{41}^{E} & S_{42}^{E} & S_{43}^{E} & S_{44}^{E} & S_{45}^{E} & S_{46}^{E}\\ S_{51}^{E} & S_{52}^{E} & S_{53}^{E} & S_{54}^{E} & S_{55}^{E} & S_{56}^{E}\\ S_{61}^{E} & S_{52}^{E} & S_{53}^{E} & S_{54}^{E} & S_{55}^{E} & S_{56}^{E} \end{array}\right]\left[\begin{array}{c} T_{1}\\ T_{2}\\ T_{3}\\ T_{4}\\ T_{5}\\ T_{6} \end{array}\right]+\left[\begin{array}{ccc} d_{11} & d_{12} & d_{13}\\ d_{21} & d_{22} & d_{23}\\ d_{31} & d_{32} & d_{33}\\ d_{41} & d_{42} & d_{43}\\ d_{51} & d_{52} & d_{53}\\ d_{61} & d_{62} & d_{63} \end{array}\right]\left[\begin{array}{c} E_{1}\\ E_{2}\\ E_{3} \end{array}\right],$$

(17) $$\left[\begin{array}{c} D_{1}\\ D_{2}\\ D_{3} \end{array}\right]=\left[\begin{array}{cccccc} d_{11} & d_{12} & d_{13} & d_{14} & d_{15} & d_{16}\\ d_{21} & d_{22} & d_{23} & d_{24} & d_{25} & d_{26}\\ d_{31} & d_{32} & d_{33} & d_{34} & d_{35} & d_{36} \end{array}\right]\left[\begin{array}{c} T_{1}\\ T_{2}\\ T_{3}\\ T_{4}\\ T_{5}\\ T_{6} \end{array}\right]+\left[\begin{array}{ccc} \varepsilon_{11}^{T} & \varepsilon_{12}^{T} & \varepsilon_{13}^{T}\\ \varepsilon_{21}^{T} & \varepsilon_{22}^{T} & \varepsilon_{23}^{T}\\ \varepsilon_{31}^{T} & \varepsilon_{32}^{T} & \varepsilon_{33}^{T} \end{array}\right]\left[\begin{array}{c} E_{1}\\ E_{2}\\ E_{3} \end{array}\right].$$

**Figure 2:**
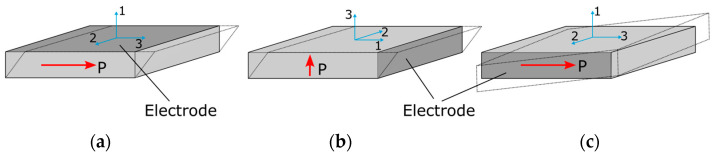
Three different modes of piezoceramic transducers used for shear wave excitation: (**a**) in-plane poled thickness-shear $d_{15}$ mode; (**b**) thickness-poled thickness-shear $d_{15}$ mode; (**c**) face-shear $d_{24}$ mode.

There are three different types of piezoceramic transducers used for shear wave excitation. These are two transducers operating in a thickness mode ($d_{15}$)—but differently poled (Figure 2a,b)—and one shear mode ($d_{24}$) transducer, as illustrated in Figure 2.

The work presented in the paper utilizes the in-plane poled thickness-shear $d_{15}$ thickness-mode transducers (Figure 2a) manufactured from the *PZT-5J* piezoceramic material. For the transducer type, poling and material used, Equations (16) and (17) can be simplified as

(18) $$\left[\begin{array}{c} S_{1}\\ S_{2}\\ S_{3}\\ S_{4}\\ S_{5}\\ S_{6} \end{array}\right]=\left[\begin{array}{cccccc} S_{11}^{E} & S_{12}^{E} & S_{13}^{E} & 0 & 0 & 0\\ S_{21}^{E} & S_{22}^{E} & S_{23}^{E} & 0 & 0 & 0\\ S_{31}^{E} & S_{32}^{E} & S_{33}^{E} & 0 & 0 & 0\\ 0 & 0 & 0 & S_{44}^{E} & 0 & 0\\ 0 & 0 & 0 & 0 & S_{55}^{E} & 0\\ 0 & 0 & 0 & 0 & 0 & S_{56}^{E} \end{array}\right]\left[\begin{array}{c} T_{1}\\ T_{2}\\ T_{3}\\ T_{4}\\ T_{5}\\ T_{6} \end{array}\right]+\left[\begin{array}{ccc} 0 & 0 & d_{13}\\ 0 & 0 & d_{23}\\ 0 & 0 & d_{33}\\ 0 & d_{42} & 0\\ d_{51} & 0 & 0\\ 0 & 0 & 0 \end{array}\right]\left[\begin{array}{c} E_{1}\\ E_{2}\\ E_{3} \end{array}\right],$$

(19) $$\left[\begin{array}{c} D_{1}\\ D_{2}\\ D_{3} \end{array}\right]=\left[\begin{array}{cccccc} 0 & 0 & 0 & 0 & d_{15} & 0\\ 0 & 0 & 0 & d_{24} & 0 & 0\\ d_{31} & d_{32} & d_{33} & 0 & 0 & 0 \end{array}\right]\left[\begin{array}{c} T_{1}\\ T_{2}\\ T_{3}\\ T_{4}\\ T_{5}\\ T_{6} \end{array}\right]+\left[\begin{array}{ccc} \varepsilon_{11}^{T} & 0 & 0\\ 0 & \varepsilon_{22}^{T} & 0\\ 0 & 0 & \varepsilon_{33}^{T} \end{array}\right]\left[\begin{array}{c} E_{1}\\ E_{2}\\ E_{3} \end{array}\right],$$

due to extrusion and crystal symmetry. The above equations explain how the piezoelectric material behaves and how the transducer can be modeled. For the transducer and poling used, the electric field is applied along the thickness of the transducer.

### 3.2. Piezoceramic Actuator Finite-Element Model

**Figure 3:**
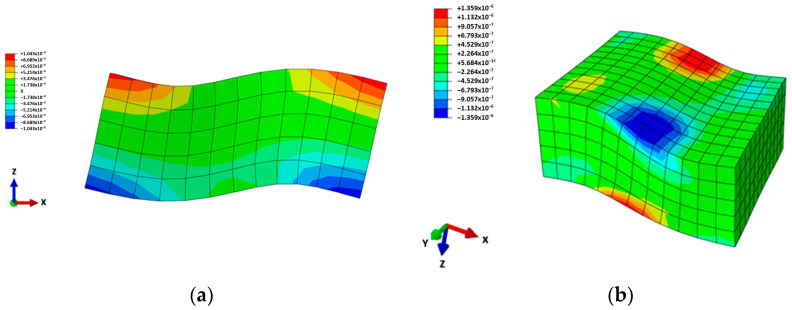
Shear wave actuator numerical simulation results after 17.5 μs: (**a**) deformation of the transducer; (**b**) strain field in the x–z plane at the bottom surface of the piezoceramic actuator.

ABAQUS was used to simulate the excitation 3-D action of a 6×6×2.5 mm *STEMiNC*^®^ shear-mode piezo plate made of the *SM411* piezoceramic material that—according to the manufacturer—is equivalent to the *PZT-5J* material [25]. The density of this material was assumed as $\rho =7800\;kg/m^{3}$. Following the strain-charge format presented in Section 3.1, the other physical properties of the material are

(20) $$S_{E}=\left[\begin{array}{cccccc} 16.2 & -4.54 & -5.9 & 0 & 0 & 0\\ -4.54 & 16.2 & -5.9 & 0 & 0 & 0\\ -5.9 & -5.9 & 22.7 & 0 & 0 & 0\\ 0 & 0 & 0 & 47 & 0 & 0\\ 0 & 0 & 0 & 0 & 47 & 0\\ 0 & 0 & 0 & 0 & 0 & 41.5 \end{array}\right]\cdot 10^{-12}\frac{m^{2}}{N}\;d=\left[\begin{array}{cccccc} 0 & 0 & 0 & 0 & 670 & 0\\ 0 & 0 & 0 & 670 & 0 & 0\\ -220 & -220 & 500 & 0 & 0 & 0 \end{array}\right]\cdot 10^{-12}\frac{C}{N}\;\frac{\varepsilon_{T}}{\varepsilon_{0}}=\left[\begin{array}{ccc} 2720 & 0 & 0\\ 0 & 2720 & 0\\ 0 & 0 & 2600 \end{array}\right]$$

where $\varepsilon_{0}=8.854\cdot 10^{-12}\frac{F}{m}$ is the permittivity in a vacuum. The transducer was excited using a 10-cycle sine tone burst signal. The signal was enveloped using the Hanning window. The central frequency and amplitude of the excitation were equal to 200 kHz and 100 *V_p-p_*, respectively. To guarantee sufficient space and time resolution for the numerical simulation stability, the following conditions were satisfied

(21) $$\Delta t=\frac{1}{20f_{max}}$$

and

(22) $$\Delta s=\frac{\lambda_{min}}{20}$$

where Δt is the time step of the simulation, $f_{max}$ is the highest frequency in the excited signal, Δs is the length of the element in the direction of the wave propagation and $\lambda_{min}$ is the shortest wavelength of interest. Thus, the transducer was modeled using 720 cubed 0.5 mm elements, where the time step was equal to $1\cdot 10^{-7}$ s. The displacement field was the output of the transducer. Examples of the numerically simulated displacement and strain fields in Figure 3 display the thickness $d_{15}$ shear mode needed to excite the SH waves.

### 3.3. Analysis of Excitation Directivity and Excitability

Once the transducer model was developed, its directivity and excitability were investigated. The directivity analysis gives spatial information regarding the direction in which the excited wave predominantly propagates. The excitability analysis identifies excitation frequencies leading to a maximum wave response.

**Figure 4:**
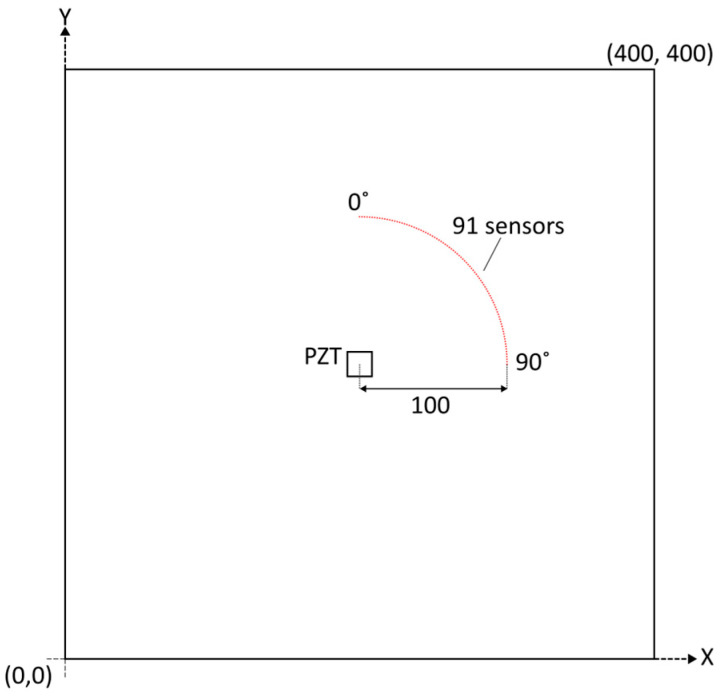
Plate model used in numerical simulations. The piezoceramic actuator that excites SH waves is positioned in the middle of the plate. Wave responses are gathered in 91 positions on the indicated quarter circle.

The 400 × 400 × 2 mm copper plate was used in the directivity and excitability analysis. The material properties of copper were assumed as follows: density, ρ = 8960 kg/m^3^; Young’s modulus, *E* = 124 GPa; and Poisson’s ratio, v = 0.34. The piezoceramic actuator—described in Section 3.2—was positioned in the middle of the plate in the numerical model. The velocities of propagating waves were gathered 100 mm from the excitation source. Altogether, 91 responses were captured on the quarter circle, as illustrated in Figure 4. This measurement configuration is sufficient to cover the entire circle due to the antisymmetry in the propagation direction of SH waves and symmetry in the propagation in the other directions.

The investigated transducer is designed to excite shear waves. However, Lamb wave excitation in the plate is also possible. Since the major focus is on the directivity and excitability of shear waves, the excitation frequency was optimized to maximize the amplitude of the SH_0_ mode. Guided wave excitation can be controlled using two parameters. These are the ratio of the transducer dimension *d* in the direction of mode propagation to its wavelength λ—marked as the width of the transducer *w* in Table 1 and referred to as the first criterion—and the ratio of the propagating mode wavelength to the transducer’s dimension perpendicular to the propagating axis—marked as the length of the transducer *L* in Table 1 [20] and referred to as the second criterion. For a given mode and frequency, the former parameter controls the energy of the transduction area of the transducer. Resonance frequencies—that lead to maximum response amplitudes—can be selected for wavelengths satisfying the equation below [20].

(23) $$\frac{n\lambda }{2}=d,$$

where *n* = 1,2,3, … The latter parameter controls the aperture and the number of generated lobes. From the far-field wave propagation theory, if the ratio λ/d is greater than or equal to one, the excitation source acts as a dipole and only the main lobe appears. In contrast, if this ratio is less than one, the aperture is reduced and side lobes of smaller amplitudes are produced. Thus, in order to generate the SH_0_ mode, the length of the transducer should be greater than (or at least equal to) the wavelength of the propagating SH_0_ mode. The amplitude of Lamb waves can be minimized by the selection of anti-resonant frequencies that satisfy the condition [20]

(24) nλ=d.

However, the amplitude of the Lamb wave was not minimized due to the square shape area of the transducer. Four different frequencies—given in Table 1—were selected for the excitation of the SH_0_ mode in the directivity analysis. These frequencies were selected to cover various scenarios with respect to the optimization criteria. In contrast, the excitability analysis covered the range from 50 to 800 kHz with increments of 25 kHz. The time step in these numerical simulations was set to $1\cdot 10^{-8}$ s to guarantee numerical stability.

**Figure 5:**
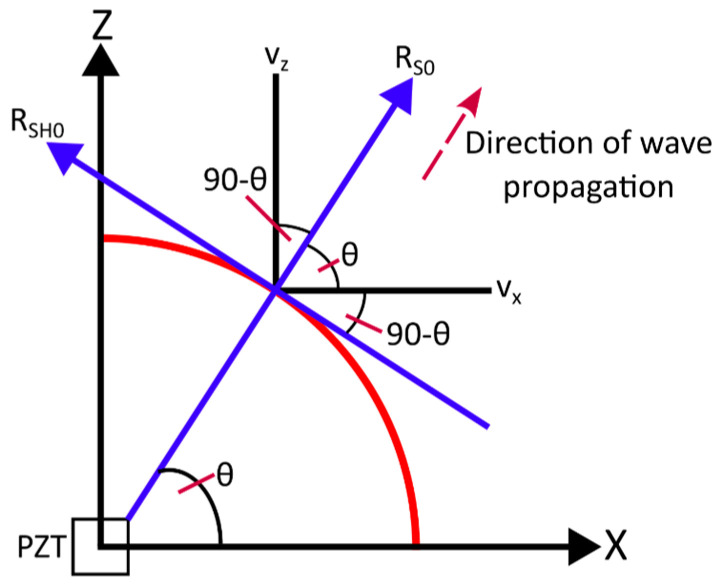
Schematic diagram explaining the geometric transformation of measurements to radial and tangential components.

The directivity analysis involved the transformation of maximum values of all response velocities into the radial and tangential components using the equations

(25) $$v_{R}=\cos\theta \cdot v_{X}+sin\theta \cdot v_{Y}\;v_{\theta }=-\sin\theta \cdot v_{X}+\cos\theta \cdot v_{Y}$$

and the geometric arrangements as illustrated in Figure 5. Then, the tangential component corresponds to the SH wave, the radial component to the S_0_ Lamb wave mode while the out-of-plane component is the A_0_ Lamb wave mode.

The procedure used for the excitation directivity involved several steps performed in MATLAB(2018a). Firstly, radial and tangential velocity components were obtained for all analyzed directions of wave propagation. Then, the Hilbert transform was applied to calculate signal envelopes and the wave packets were identified using dispersion curves. Finally, the enveloped wave packet maxima were arranged in polar plots. These directivity plots give wave amplitudes for all angles of propagation, with respect to the excitation source.

## 4. Numerical Simulation Results

This section presents numerical simulation results. The excitation directivity plots are given first. The results are presented for one, two and three actuators. Then, the excitability results are presented for one actuator.

### 4.1. Excitation Directivity

**Figure 6:**
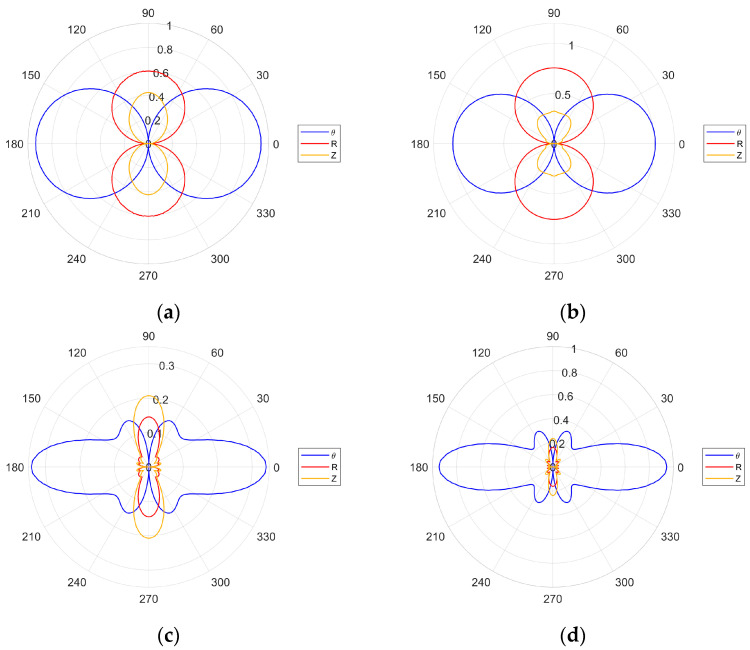
Numerically simulated directivity patterns for various excitation frequencies: (**a**) 162.5 kHz; (**b**) 189.5 kHz; (**c**) 378.5 kHz; (**d**) 454.5 kHz.

The directivity plots for the selected excitation frequencies are given in Figure 6. The plots in Figure 6a,b—for the 162.5 and 189.5 kHz excitation frequencies—display only the main lobes for the SH_0_ wave. This is in line with the second optimization criterion—given by Equation (24)—that is not fulfilled. In contrast, for the 378.5 and 454.5 kHz excitation frequencies, the second criterion is fulfilled and the results in Figure 6c,d display narrower main lobes with clearly visible side lobes.

**Figure 7:**
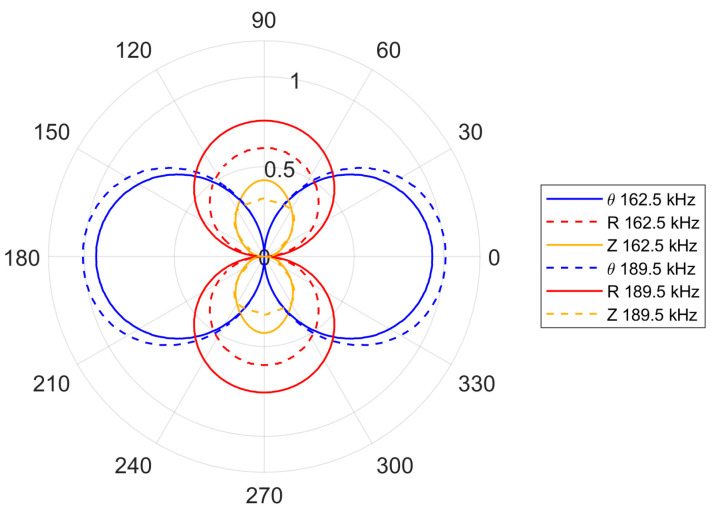
Amplitude comparison simulated plots for the 162.5 and 189.5 kHz excitation frequency.

There is also a noticeable difference in the amplitudes of the S_0_ and A_0_ modes, between the cases where the wavelength is equal to (Figure 6c) and shorter than (Figure 6d) the transducer wavelength. For the 189.5 kHz excitation frequency, the first optimization criterion—given by Equation (23)—is fulfilled. Thus, the amplitude of the SH_0_ wave should be higher than for the 162.5 kHz excitation frequency. This is illustrated in Figure 7, where the amplitudes of the first two excitation frequencies are compared. None of the four excitation cases investigated were tailored to the Lamb wave optimization criterion and the evidence of this is in Figure 6 and Figure 7.

**Figure 8:**
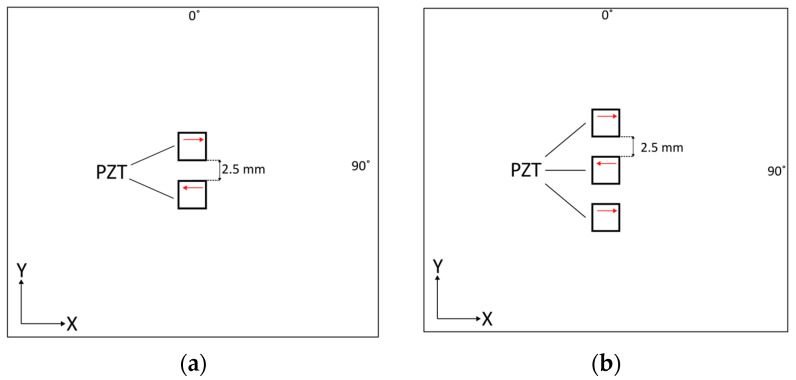
Numerical simulation set-up for the multiple-transducer transducer excitation of the SH_0_ mode: (**a**) two transducers; (**b**) three transducers. The red arrows indicate the directions of oscillation.

The analysis of geometrical properties of the investigated transducer indicates that the 454.5 kHz frequency was optimal for SH_0_ mode excitation. Therefore, this frequency was selected for the analysis of excitation with two and three identical transducers to see whether the multiple-transducer configuration improves the results. Multiple transducers were separated by the distance of 2.5 mm. This distance is equal to half of the wavelength of the SH_0_ mode (5 mm). For the two-transducer configuration, the transducers were oscillating in the opposite direction in order to be in phase. In order to achieve this in-phase action, the outer transducers operated in one direction and the inner transducer in the opposite direction for the three-transducer configuration. Both multiple-transducer configurations are illustrated in Figure 8.

**Figure 9:**
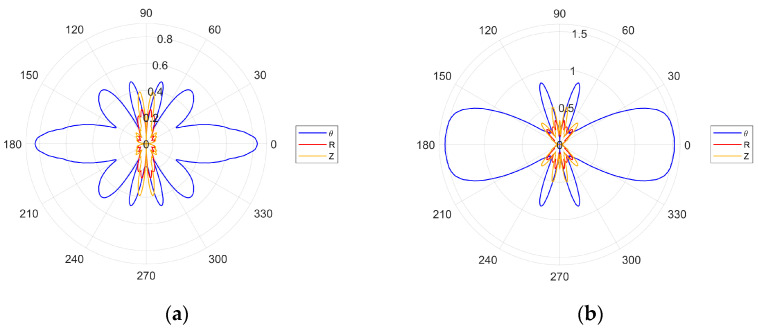
Simulated directivity plots for the multiple-transducer excitation configuration: (**a**) two transducers; (**b**) three transducers.

**Figure 10:**
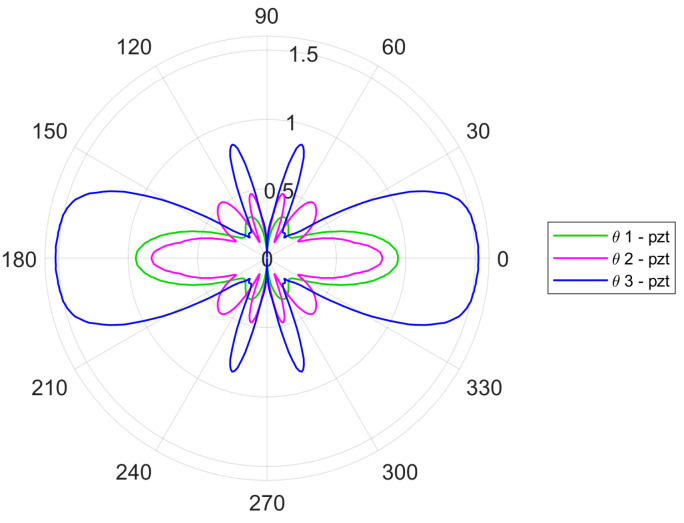
Comparison of the SH_0_ amplitude for single-, double- and triple-transducer excitation configurations.

Figure 9 gives the excitation directivity plots for the analyzed multiple-transducer configurations. The main lobe for the two-transducer configuration in Figure 9a is thinner than the main lobe for the three-transducer configuration in Figure 9b. Both plots exhibit more side lobes if compared with the results presented in Figure 6. However, the three-transducer configuration in Figure 9b displays less side lobes that are, in addition, thinner. Figure 10 compares the amplitudes of single- and multiple-transducer configurations. Here, only the amplitude of SH_0_ is displayed for clarity. Interestingly, the results for the single-transducer configuration are better than for the two-transducer configuration with respect to the higher amplitude of the main lobe and smaller number of side lobes. However, the three-transducer configuration outperforms the other two configurations since the amplitude of the main lobe is much larger and the number of side lobes is not significantly better than for the two-transducer and not significantly worse than for the single-transducer configurations.

### 4.2. Excitability

The excitability of the analyzed piezoceramic actuator is investigated using two measuring points located 100 mm from the excitation source at 0° and 90° angle to the direction of the SH wave propagation. The former location displays the maximum amplitude of the SH wave, whereas the latter exhibits the maximum amplitude of the Lamb wave. A Hanning window-modulated sine tone burst was used for the excitation. The number of cycles varied with the frequency of excitation in order to keep the window length constant as $1\cdot 10^{-4}$ s. The velocity signal responses were collected for the x, y and z directions and converted to relevant velocity components for different wave modes. The SH_0_ wave was captured at a 90° angle to the direction of the wave propagation, whereas the S_0_ and A_0_ Lamb wave modes were captured at 0° relative to the same direction.

**Figure 11:**
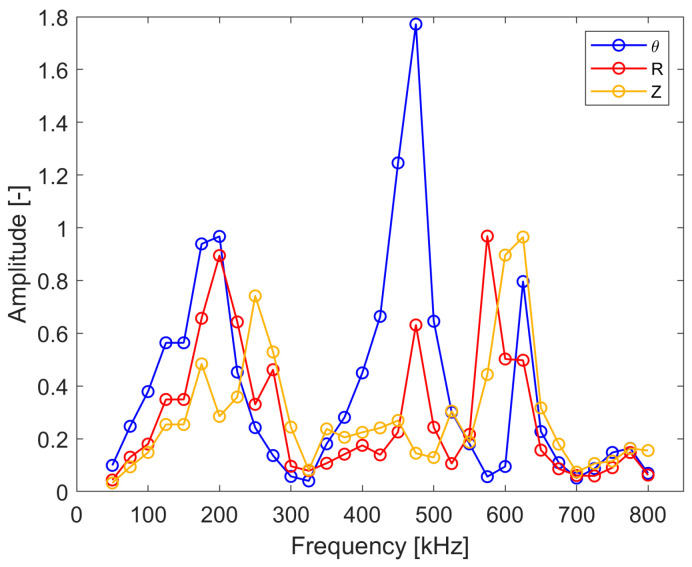
Simulated excitability curves for the analyzed copper plate.

Figure 11 gives the excitability curves for the analyzed copper plate. The results show that the amplitude of excitation varies significantly with the excitation frequency for the analyzed wave modes. However, three significant peaks can be observed in these characteristics for the frequencies equal to approximately 189.5, 475 and 625 kHz. The first frequency corresponds well to the excitation frequency selected following the optimization criteria. However, the other frequencies do not correspond to the previously selected excitation frequencies. This suggests that the geometry of the transducer is not the only factor that influencing the amplitude of excitation. The largest peak for the SH_0_ wave mode can be observed for 475 kHz excitation. This frequency also complies well with the second (aperture) criterion and therefore could be used for effective shear wave excitation.

**Figure 12:**
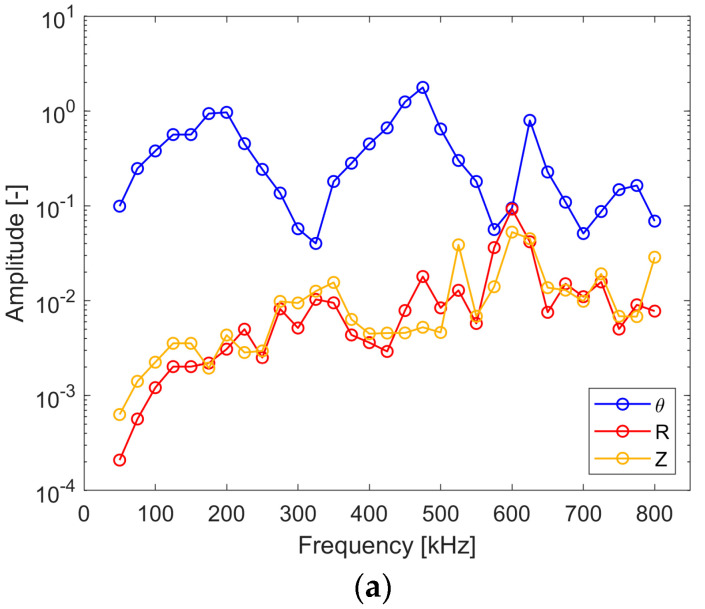

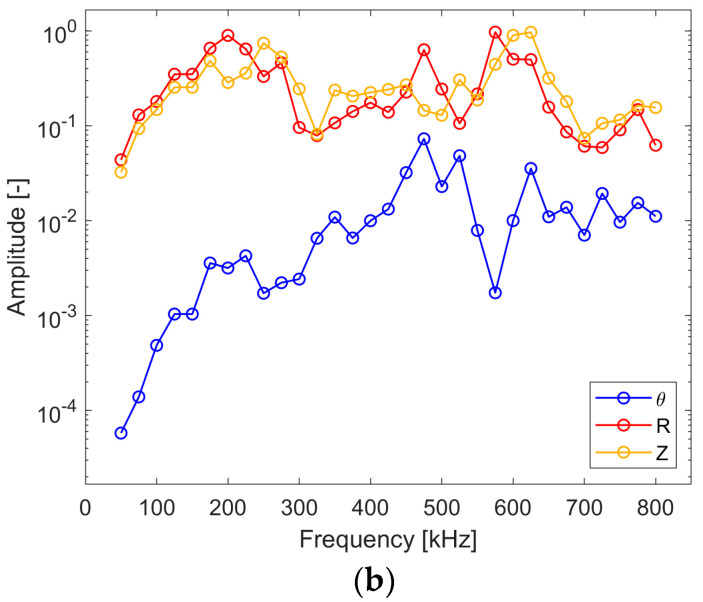
Simulated excitability curves for the wave modes captured in one point where (**a**) SH_0_ wave propagates (0°); (**b**) S_0_ wave propagates (90°).

Figure 12 gives the excitability characteristics for all wave modes (i.e., SH_0_, A_0_ and S_0_) captured in the same point. This arrangement allows one to see what the amplitude of the Lamb wave modes is in the direction of the SH_0_ wave propagation and vice versa, i.e., what the amplitude of the SH_0_ mode is in the direction of the S_0_ wave mode propagation. One should note that a logarithmic amplitude scale is used here due to large amplitude differences. The results in Figure 12 are in line with the directivity patterns, as expected. The SH_0_ wave exhibits the largest amplitude for the 0° angle direction and the S_0_, A_0_ wave modes display the largest amplitude for the 90° angle direction. The good news is that the differences in the amplitudes between the analyzed wave modes is significant.

## 5. Experimental Tests

Experimental tests were performed to validate selected results. This section describes the work undertaken. The experimental arrangements are described first. Then, directivity and excitability plots are obtained.

### 5.1. Experimental Arrangements

**Figure 13:**
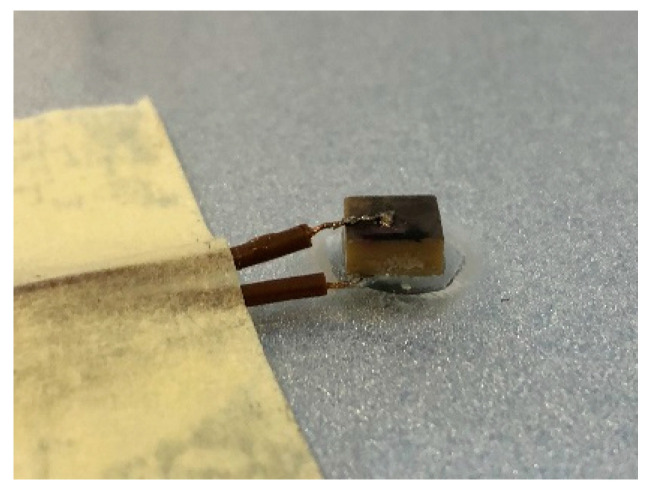
Transducer bonding on the copper plate. The electrodes are located on both sides of the transducer.

**Figure 14:**
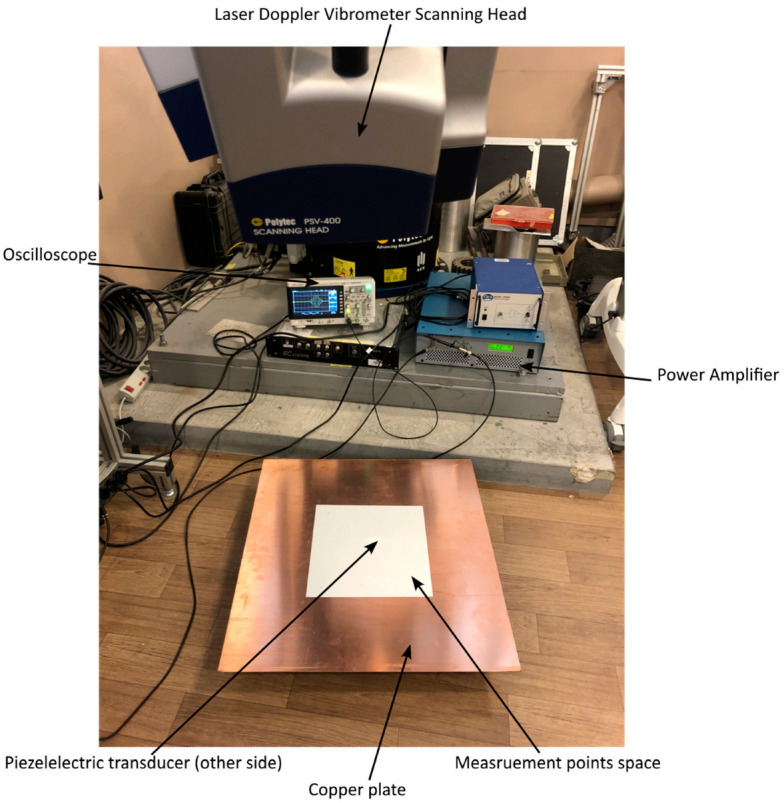
Experimental set-up used to obtain directivity and excitability curves.

The STEMiNC^®^ shear mode piezo actuator—modeled in Section 3 and utilized in subsequent numerical simulations in Section 4—was used in the experimental tests. This low-profile actuator was surface-bonded to a rectangular 800 × 800 × 1 mm copper plate using cyanoacrylate epoxy. The PZT actuator was originally supplied with nickel electrodes bonded on the top and bottom surfaces. Therefore, perfect transducer bonding on the plate was not possible as shown in Figure 13. Excitation signals were generated using a Keysight 33500B Series Waveform Generator and amplified using an E&I 200 W, AB class amplifier. The frequency of excitation was equal to 162.5, 189.5, 378.5 and 454.5 kHz, following the same pattern of frequencies used in numerical simulations. For the first three frequencies, a ten-cycle Hanning-windowed tone burst was used for excitation. The largest excitation frequency utilized fifteen cycles in the tone bursts. For all signals, the amplitude was set to 100 V_pp_. Signal responses were gathered using a Polytec PSV-400-3-D laser scanning vibrometer system. The area of the plate where the measurements were undertaken was covered by a refractive film to ensure a good signal-to-noise ratio of responses. Figure 14 shows the experimental arrangements.

Wave responses at 180 points were gathered in three x, y, z directions. Due to the symmetry, these points were located on a half-circle of 100 mm radius and spaced by 1°. Data pre-processing based on band-pass filtering was used to improve the signal-to-noise ratios. The pass-band frequencies were ±20 kHz from the central excitation frequency. The stop-band frequencies were equal to ±40 kHz with the attenuation of 80 dB. For the transducer excitability patterns, the excitation frequency covered the range of 50–800 kHz. The measurements were taken with the 25 kHz increment. For each excitation frequency, a set of four measuring points were used at angles of 0°, 90°, 180° and 270°, following the same procedure as in numerical simulations.

### 5.2. Experimental Results

#### 5.2.1. Transducer Directivity

**Figure 15:**
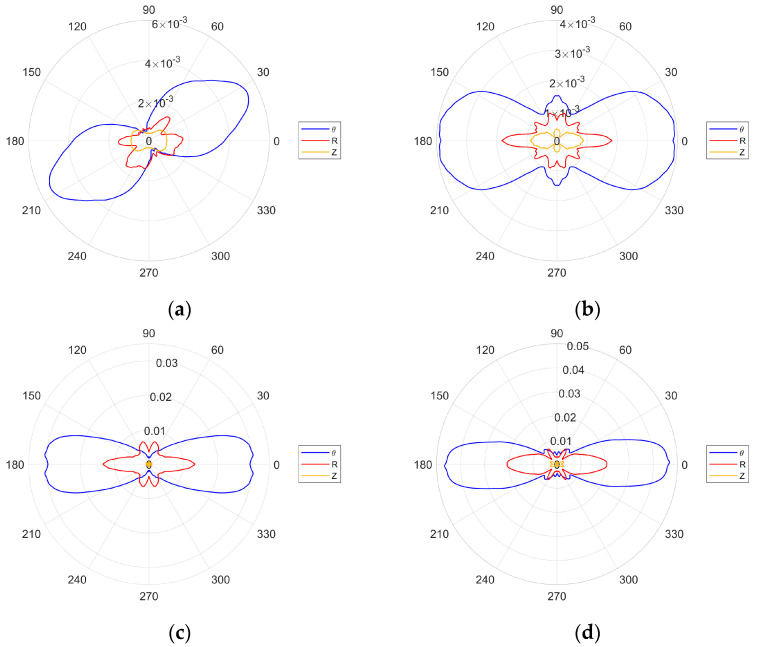
Experimental transducer directivity patterns for: (**a**) 162.5 kHz; (**b**) 189.5 kHz; (**c**) 378.5 kHz; (**d**) 454.5 kHz.

**Figure 16:**
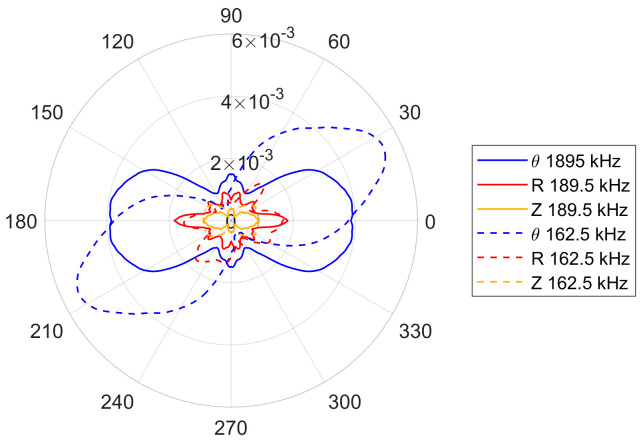
Comparison of amplitudes of transducer directivity patterns for 162.5 and 189.5 kHz excitation frequency.

For the measurements taken on a half-circle, a full directivity pattern can be obtained using the symmetry and antisymmetry of the SH_0_ and Lamb wave modes. For each individual characteristic, the symmetry lines were located at 0–180° and 90–270°. However, in the case of the lowest frequency considered—162.5 kHz—the symmetry line had to be transferred to 120–300°, which was the result of the obtained preliminary measurement. Figure 15 presents these mirrored patterns for all analyzed frequencies, whereas Figure 16 compares the amplitudes for the two lowest excitation frequencies analyzed. At first glance the experimental results in Figure 15 and Figure 16 look similar to the simulated results in Figure 6 and Figure 7. However, some discrepancies can be observed. For the two lower excitation frequencies—for which the first criterion is fulfilled and the second criterion is not—one would expect to see only two main lobes for the SH_0_. This can be observed for the 162.5 kHz frequency but not for the 189.5 kHz, where very small—but distinguishable—additional lobes can be seen alongside the 90–270° axis. The opposite can be observed for the two higher frequencies, where the lobes on the line 90–270° either do not appear for the SH_0_ wave or are very small. Nevertheless, both smaller excitation frequencies in Figure 15a,b exhibit wider lobes than larger excitation frequencies in Figure 15c,d, as expected.

The effect of the second criterion can be seen in Figure 16, which compares the amplitudes of the relevant characteristics. The results show that the relevant amplitudes are quite similar, suggesting that the excitation frequency has no influence on the amplitude. However, this contradicts the theory. The only explanation could relate to the position of the electrodes. The pattern for the 162.5 kHz is produced at the angle orthogonal to the position of the electrodes and this could somehow magnify the relevant amplitude. The results also show that the S_0_ Lamb wave modes are in line with the SH_0_ modes, as expected.

In summary, the experimental results show that the transducer bottom–top configuration has a significant influence on the directivity and amplitude of excitation. Nevertheless, for three out of the four frequencies investigated, the results confirm the findings from numerical simulations to some extent.

#### 5.2.2. Transducer Excitability

**Figure 17:**
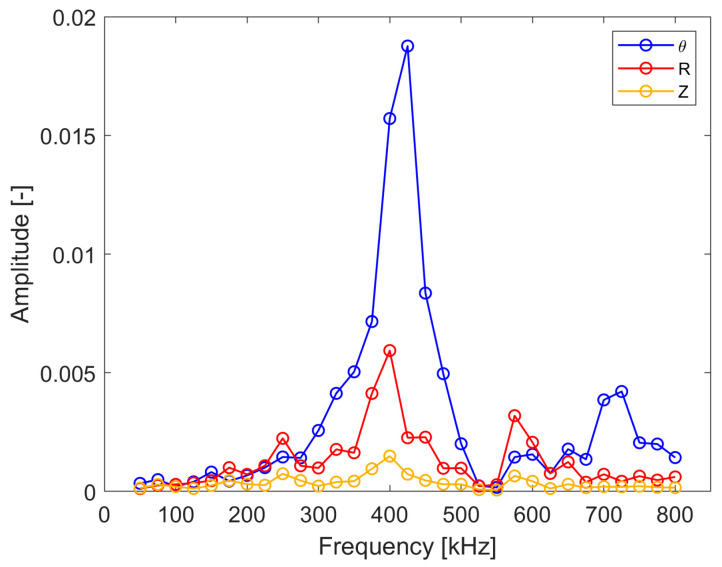
Experimental transducer excitability characteristics for the SH_0_ and fundamental Lamb wave modes.

**Figure 18:**
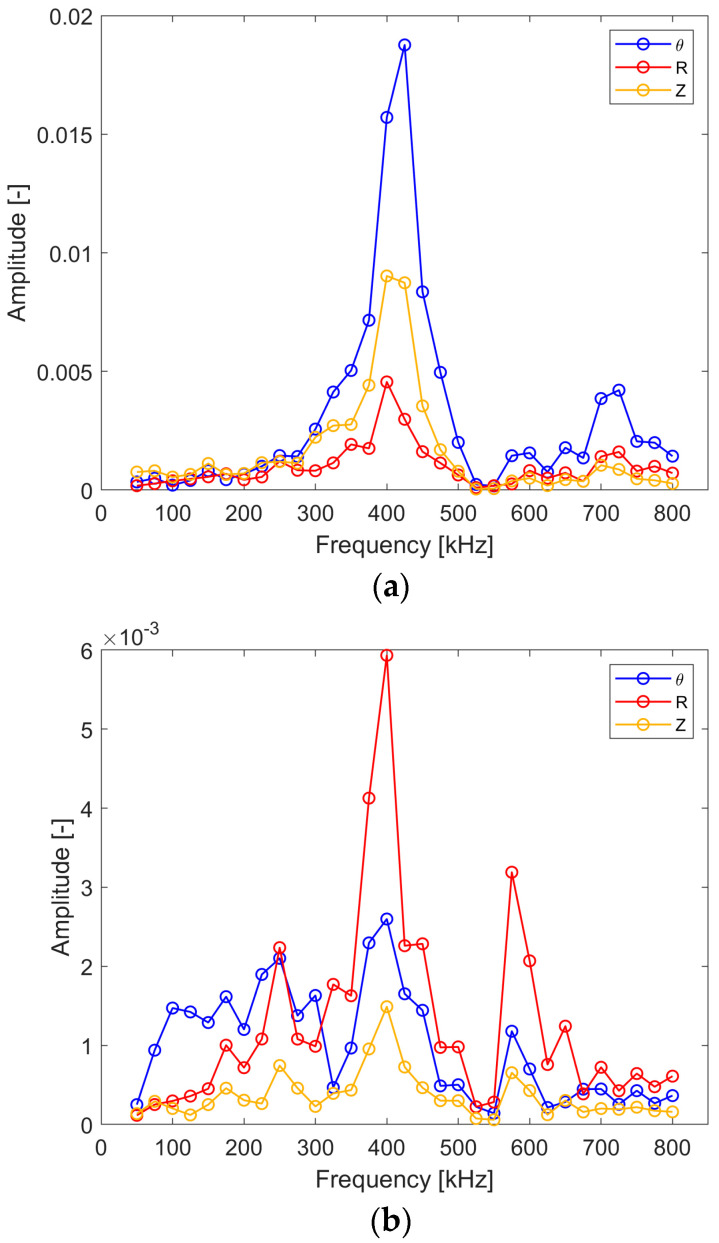
Experimental excitability curves for the wave modes captured in one point where (**a**) SH_0_ wave propagates (0°); (**b**) S_0_ wave propagates (90°).

The results for the transducer excitability are presented in Figure 17 and Figure 18. The results in Figure 17 display one predominant peak around 405–425 kHz that corresponds well to the resonance of the transducer and is in line with the numerical simulation results.

Following the procedure used in numerical simulations, Figure 18 gives the excitability characteristics for all wave modes (i.e., SH_0_, A_0_ and S_0_) captured at the same point in order to see what the amplitude of the Lamb wave modes is in the direction of SH_0_ wave mode propagation and vice versa. The results are similar to the numerical simulation results qualitatively. The amplitude difference between the analyzed shear and Lamb wave modes is significant but not to the extent shown by the numerical simulations. Nevertheless, for most of the excitation frequencies in the analyzed range, the amplitude difference seems to be large enough to perform reliable analysis based on shear waves.

#### 5.2.3. Directivity Analysis near the Resonance Frequency of the Transducer

**Figure 19:**
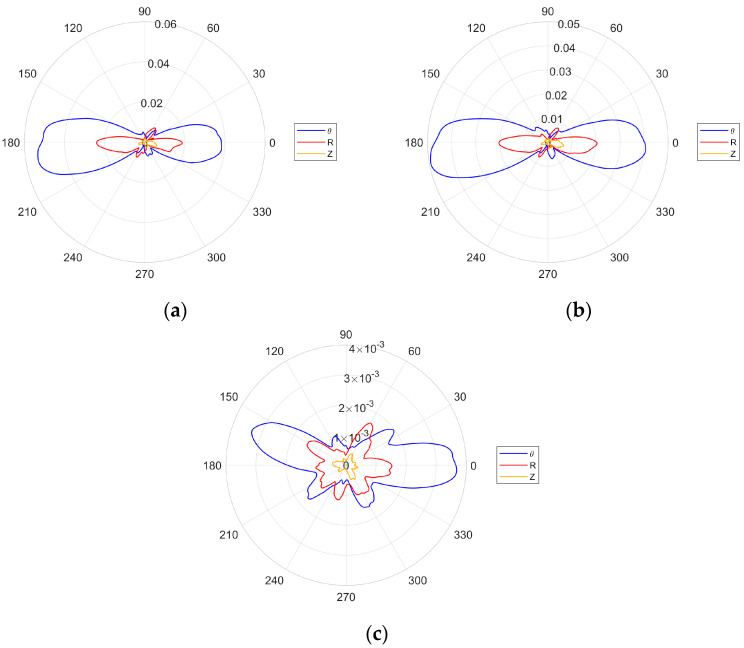
Experimental transducer directivity patterns for excitation frequencies corresponding to maximum amplitudes of SH_0_: (**a**) 405 kHz; (**b**) 425 kHz; (**c**) 700 kHz.

Following the results presented in Section 5.2.1 and Section 5.2.2, another set of directivity experiments was undertaken for the excitation frequencies in the vicinity of the transducer’s resonance. In contrast to the analysis presented in Section 5.2.1, this time a pattern of 360 measurements (spaced by 1° angle) were taken on a full circle of 100 mm radius. Three different excitation frequencies were selected in these experiments, i.e., 405, 425 and 700 kHz. The first two frequencies correspond to the maxima levels of the SH_0_ and S_0_ Lamb wave modes. For 475 kHz, the amplitude of SH_0_ is still relatively high, whereas the amplitude of S_0_ is close to zero. The results are presented in Figure 19. With all three chosen frequencies having smaller wavelengths than the transducer length, the characteristics patterns have quite a narrow range with a varying number and amplitude of side lobes. This corresponds well with the geometric analysis and numerical simulations. The results also exhibit a nonuniform amplitude distribution between the left and right half of the characteristics. The difference between the amplitudes can be also observed. Both effects correspond well with the transducer bonding problem explained in Section 5.2.2. The results also show that the excitation of a single SH_0_ mode is not possible since the ratio of the SH_0_ to S_0_ amplitudes is very similar for all selected frequencies.

## 6. Conclusions

The directivity and excitability of the *STEMiNC*^®^ *SM411* shear mode piezo actuator has been investigated. The work has involved theoretical analysis, numerical simulations and experimental validation. The focus was on the directivity and excitability of the transducer. The latter was of particular interest. Modeling and numerical simulations were more successful than experimental validation to achieve the objective of the paper. Nevertheless, the experiments performed validated numerical simulations to some extent. The major conclusions from this work can be summarized as follows.

The results confirm that the transducer investigated generates not only the desired shear wave modes but also undesired Lamb wave modes, as expected. Therefore, transducer design and modeling are important in order to maximize the excitation of the former wave and minimize the excitation of the latter waves. The theoretical geometric criteria used for transducer modeling were very useful to achieve this task and obtain directivity characteristics that exhibit two main lobes of shear waves and geometry/frequency-dependent amplitudes. However, the results also show that in practice other elements than geometric criteria also affect excitation amplitudes. Therefore, the geometric criteria are more useful for the directivity than for the excitability analysis of the analyzed transducer. As for the actuator excitability, the most effective frequency is the resonance frequency of the actuator. However, the response amplitude for this frequency differs from the one obtained using geometric properties of the transducer. Nevertheless, the simulated and experimental excitability characteristics show that amplitudes of excited shear waves are always significantly larger than amplitudes of Lamb waves. The preliminary simulated results also indicate that the amplitude characteristics of excitation could be improved when more three—instead of one—actuators are used for excitation. However, more simulated work and experimental tests are needed to confirm this finding.

Experimental investigations show that transducer uniform bonding is very important for directivity and excitability. Both characteristics are significantly deteriorated if the uniform bonding is not achieved. However, for the selected transducer, uniform bonding was very difficult—if not impossible—to achieve due to the top–bottom surface electrode configuration. As a result, the directivity patterns were skewed and the amplitude in the direction orthogonal to the electrodes was magnified. Therefore, one should be very careful when the symmetry/antisymmetry of wave propagation and mirroring of the half-circle pattern is used to present a full-circle directivity pattern of the transducer.

In summary, when the selected actuator is used for shear excitation, the best solution is to tailor the transducer in such a way that at the resonant frequency the desired directivity is achieved.

Future work in this area should focus on better models of surface-bonded transducers. Transducer properties and the uniformity of bonding should be investigated in more detail. In addition, more work is needed on shear work excitation based on multiple-actuator configurations.

## Figures and Tables

**Table 1:** Frequencies selected for the excitation of SH_0_ wave, following the optimization criteria. The “✓” sign indicates that the relevant criterion is fulfilled.

Excitation Frequency [kHz]	First Criterion $w=\frac{n\lambda }{2}$	Second Criterion $\frac{\lambda }{L}\leq 1$
162.5	X	X
189.5	✓	X
378.5	X	✓ (λ\L=1)
454.5	X	✓ (λ\L<1)

## Data Availability

All simulated and experimental data are available upon request.
